# The neurology of rhizomelic chondrodysplasia punctata

**DOI:** 10.1186/1750-1172-8-174

**Published:** 2013-10-30

**Authors:** Annemieke M Bams-Mengerink, Johannes HTM Koelman, Hans Waterham, Peter G Barth, Bwee Tien Poll-The

**Affiliations:** 1 Department of Pediatric Neurology/Emma Children’s Hospital, Academic Medical Center, University of Amsterdam, Amsterdam, The Netherlands; 2 Department of Neurology, Academic Medical Center of Amsterdam, Amsterdam, The Netherlands; 3 Laboratory of Genetic Metabolic Diseases, Academic Medical Center of Amsterdam, Amsterdam, The Netherlands; 4 Meibergdreef 9, Amsterdam 1105 AZ, The Netherlands

**Keywords:** Rhizomelic chondrodysplasia punctata, Skeletal dysplasia, Peroxisome, Plasmalogen, Intellectual disability, Epilepsy, Evoked potentials

## Abstract

**Background:**

To describe the neurologic profiles of Rhizomelic chondrodysplasia punctata (RCDP); a peroxisomal disorder clinically characterized by skeletal abnormalities, congenital cataracts, severe growth and developmental impairments and immobility of joints. Defective plasmalogen biosynthesis is the main biochemical feature.

**Methods:**

Observational study including review of clinical and biochemical abnormalities, genotype, presence of seizures and neurophysiological studies of a cohort of 16 patients with RCDP.

**Results:**

Patients with the severe phenotype nearly failed to achieve any motor or cognitive skills, whereas patients with the milder phenotype had profound intellectual disability but were able to walk and had verbal communication skills. Eighty-eight percent of patients developed epileptic seizures. The age of onset paralleled the severity of the clinical and biochemical phenotype. Myoclonic jerks, followed by atypical absences were most frequently observed. All patients with clinical seizures had interictal encephalographic evidence of epilepsy. Visual evoked (VEP) and brain auditory potential (BAEP) studies showed initial normal latency times in 93% of patients. Deterioration of VEP occurred in a minority in both the severe and the milder phenotype. BAEP and somatosensory evoked potentials (SSEP) were more likely to become abnormal in the severe phenotype. Plasmalogens were deficient in all patients. In the milder phenotype levels of plasmalogens were significantly higher in erythrocytes than in the severe phenotype. Phytanic acid levels ranged from normal to severely increased, but had no relation with the neurological phenotype.

**Conclusion:**

Neurodevelopmental deficits and age-related occurrence of seizures are characteristic of RCDP and are related to the rest-activity in plasmalogen biosynthesis. Evoked potential studies are more likely to become abnormal in the severe phenotype, but are of no predictive value in single cases of RCDP.

## Background

**Figure 1:**
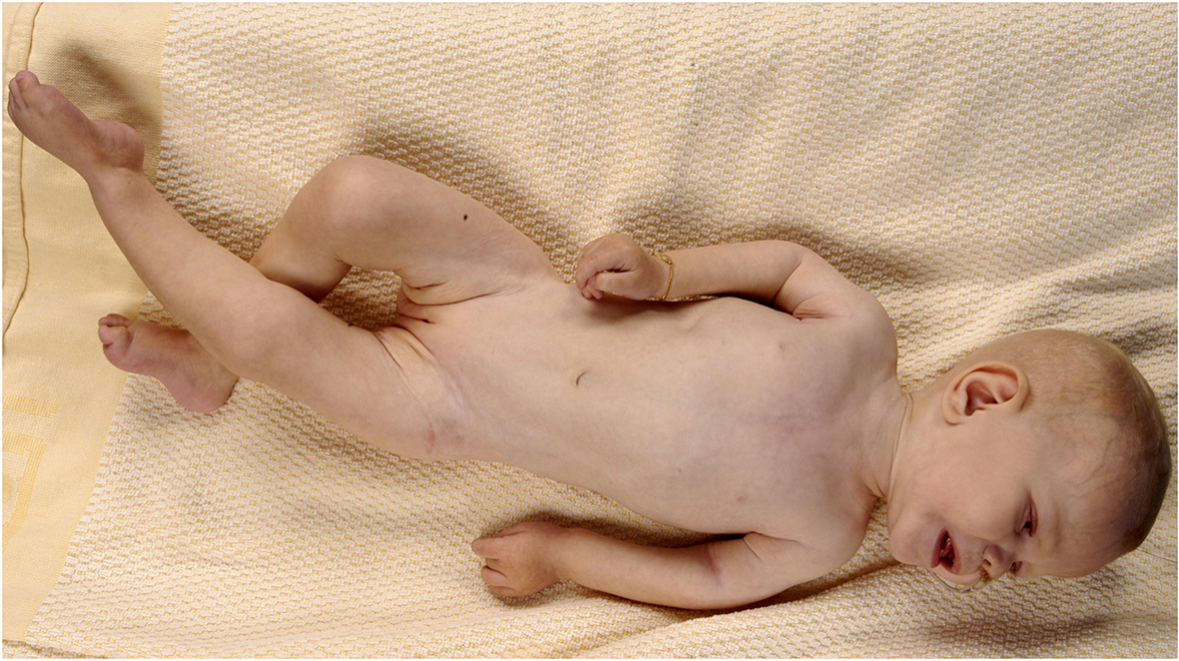
**A patient with RCDP type 1.** Anterior aspect of a patient with the severe phenotype at age 4 years. The clinical phenotype of RCDP is characterized by facial dysmorphic features, bilateral rhizomelia and arthrogenic contractures. Growth is severely impaired. Patients achieve hardly any developmental milestones.

Rhizomelic chondrodysplasia punctata (RCDP) is a rare disorder of peroxisomal metabolism, with an estimated incidence of 1:100.000. There are 3 genetic subtypes [1]. RCDP type 1 (OMIM 215100), caused by mutations in the *PEX7* gene, is the most common type [2]. RCDP type 2 (OMIM 222765) and 3 (OMIM 600121) are single enzyme deficiencies in the plasmalogen biosynthesis pathway [3]. Clinically the 3 genetic subtypes are indistinguishable; within each subtype there is a wide variety in severity of the disease [4]. At the severe end of the spectrum, the disease is characterized by a typical facial appearance, congenital cataracts, rhizomelia, transient periarticular calcifications, arthrogenic contractures, somatic growth impairment and near absence of developmental milestones (Figure 1) [5]. Magnetic Resonance Imaging (MRI) of the brain shows both developmental and regressive abnormalities, including delayed meylination, increased ventricular size and subarachnoidal spaces, supratentorial myelin abnormalities, progressive cerebellar atrophy and cervical stenosis [6]. Life expectancy is dramatically shortened. In patients with the milder variant, skeletal problems are mild. Mild RCDP may be suspected when a combination of congenital cataracts and developmental delay is found [7]. The biochemical hallmarks of RCDP are: 1) decreased concentration of plasmalogens in erythrocytes in all three subtypes [8] and 2) high levels of phytanic acid, which accumulates in an age and diet dependent way in RCDP type 1. The severity of decreased erythrocyte plasmalogens corresponds with the severity of the disease and abnormalities seen on MRI of the brain [6].

Since plasmalogens are important constituents of neuronal membranes, it is assumed that the lack of plasmalogens results in abnormal excitatory activity of neurons in the central nervous system (CNS) and in abnormal nerve conduction in both the central and peripheral nervous tissue [9]. We therefore reviewed the medical records of 16 patients with RCDP, with emphasis on the occurrence of seizures, and related electrophysiological data to clinical and biochemical parameters within the clinical spectrum and natural history of RCDP.

## Methods

We retrospectively reviewed the medical records and clinical neurophysiological assessments of 16 patients with RCDP known to the reference centre for peroxisomal disorders in the Netherlands. The diagnosis was confirmed by biochemical analysis, e.g. RBC plasmalogen levels and plasma phytanic acid levels, and genetic testing, as previously described [10-13]. Clinical data were obtained by review of clinical histories and own observations. We rated patients as either having the severe or the milder form of RCDP, based on their ability to walk independently and verbal communication skills. Neuroradiological features of ten of the patients reported here have been reported previously [6]. The diagnosis of epilepsy was based on the physicians’ assessment of a clinical history of seizures. Semiology, age at onset, frequency of occurrence and treatment of seizures were recorded. Neurophysiologic investigations were conducted by a certified technician at the AMC. Data interpretation was performed by one clinical neurophysiologist (JHTMK). All records were assessed for quality with respect to configuration and reproducibility.

Ethics approval: Medical- and neurophysiological records were obtained retrospectively (using patient files) or to ensure full patient care (considering we noticed that several of our patients had epilepsy).

### Electro encephalography (EEG)

EEGs were recorded with video-monitoring and according to the 10–20 system for at least 30 minutes. All EEG’s were rated on three features: background pattern, epileptic discharges, and focal abnormalities.

### Visual-evoked potentials (VEP)

Stroboscopic flash stimuli were presented, while awake, monocularly at a distance of 1.45 m with a stimulation rate of 1.3 Hz. Two series of 100 stimuli presented to each eye were recorded, superimposed with a Nicolet Viking IV unit and checked for reproducibility. Recording electrodes were placed over the midocciput and 5 cm laterally on O1 and O2, and referenced to C_z_. Electrode impedances were less than 5 kΩ. The bandpass was 0.16–80 Hz, and the time of analysis was 250 ms. The P100 latencies were determined.

### Somatosensory-evoked potentials (SSEP)

Median nerve SSEPs were recorded with standard procedures [14]. The left or right median nerve was stimulated electrically at the wrist with surface electrodes at a frequency of 3.1 Hz and stimulus duration of 0.2 mec. Central conduction time (CCT) was calculated by subtracting the latency of N13 to N20.

### Brainstem auditory-evoked potentials (BAEP)

Rarefaction clicks with an intensity of 75 dB above the hearing threshold with a maximum of 95 dB were delivered. The frequency of stimulation was 11 Hz. Stimuli were presented monaurally with contra lateral masking using a noise 40 dB above threshold. Two series of 2000 potentials were recorded, averaged, and superimposed with a Nicolet Viking IV (Nicolet Instruments Corporation, Madison, WI, USA). BAEPs were recorded with surface electrodes over the mastoids and referenced to C_2_. Electrode impedances were less than 5 kΩ, the bandpass was 0.16–3.2 kHz, and the time of analysis was 10 ms. Latencies of wave I to V and I-III and I-V intervals were measured.

The BAEP, VEP, and SSEP studies were all reviewed by an independent observer (JHTMK) and scored as: (1) normal; (2) mildly abnormal (+ 2–4 SD); and (3) markedly abnormal (>4 SD), based on our references.

## Results

In this cohort 15 patients had RCDP type 1 (*PEX7*) and one patient had RCDP type 2 (*GNPAT, glyceronephosphate O-acyltransferase*). Nine patients with the severe phenotype were homozygous for the most common mutation (L292X) in *PEX7*. The missense mutation H285R was seen in the patients with the milder phenotype. In 2 only one mutation in *PEX7* was found. In patient 5 gene expression studies revealed a homozygous expression of the heterozygous mutation at L292X. The patient with RCDP type 2 had a severe phenotype similar to the patients with RCDP type 1.

Duration of follow up ranged from 7 months to 30 years (median 8.3 years, mean 10.8 years). Twelve patients were severely affected, whereas 4 had a milder phenotype. Patients with the severe phenotype only reached the earliest developmental milestones. Minor improvements in social interaction, awareness of their surroundings and grabbing with their fingers were present. The majority of patients required tube-feeding, because of impaired sucking and swallowing. At neurological examination visual responses were brief despite of cataract extraction and aphakia correction. There was axial hypotonia and spastic tetraparesis, with increased tendon reflexes and positive Babinski signs.

In patients with the milder phenotype delayed motor development was noticed in the second half of the first year. Patient 14 attained the ability to walk independently at age 1.5 year; the other 3 patients (patients 13, 15, 16) were over 2 years (range 2–8 yrs). Speech and language development was also delayed. Patients were able to feed themselves. All 4 patients attended schools for children with special needs. Patients who reached adulthood (patient 15, 16) needed round-the-clock care facility. With increasing age they lost ambulation ability, mostly due to arthrogenic contractures. The neurological examination was characterized by intellectual disability, contractures of the large joints and scoliosis. Pyramidal tract signs were present in patient 16. Table 1 depicts clinical (including MRI findings [6]) and biochemical characteristics of this cohort. Patients with the milder phenotype had significantly higher levels of plasmalogens in erythrocytes. Phytanic acid levels in plasma varied.

**Table 1:** Patient characteristics and peroxisomal test results

**Patient**	**Disease severity**	**Genotype mutations, cDNA**	**% C16:0DMA/C16:0♦ (normal 6.8–11.9)**	**% C18:0DMA/C18:0 ♦ (normal 10.6–24.9)**	**Phytanic acid♦ (normal <10 umol/l)**	**Age (mo) cataract extraction**	**Best developmental performance**	**Ages (mo) at MRI and findings**	**Remarks**
1	Severe	*PEX 7*:c [875 T > A]; [875 T > A]	nd	nd	10	7	Smiles responsively, no intentional movement, gastrostomy fed	24: Wide extra cerebral liquor spaces; ventricular enlargement; cerebellar atrophy; abnormal white matter signal intensity.	Ref [6] pt 1
									† 4 yrs 11/12
2	Severe	*PEX 7*:c [875 T > A]; [875 T > A]	1.2	0.5	6.4	3	Smiles responsively, spontaneous movement of fingers, bottle/spoon fed	23 and 48: Delayed myelination, abnormal white matter signal intensity; cerebellar atrophy. Cervical stenosis.	Ref [6] pt 2.
3	Severe	*PEX 7*:c [875 T > A]; [875 T > A]	0	0	12.4	3	Smiles responsively, turns from back to belly, gastrostomy fed	10, 22 and 60: Delayed myelination; abnormal white matter signal intensity; progressive cerebellar atrophy. Narrow cervical canal.	Ref [6] pt 3.
4	Severe	*PEX 7*:c [875 T > A]; [875 T > A]	0.2	0.2	4.4	3	Smiles responsively, no intentional movement, gastrostomy fed	2 and 16: Delayed myelination; abnormal white signal intensity; cerebellar atrophy; progressive ventricular enlargement. Progressive narrowing thoracic spinal canal.	Ref [6] pt 5.
									† 1 yr 5/12
5	Severe	*PEX 7*:c [875 T > A];?	1.6	2.9	25.2	5	Smiles responsively, vocalizes, spontaneous movement of fingers, gastrostomy fed	10, 27 and 48: Delayed myelination; abnormal white signal intensity; cerebellar atrophy; progressive ventricular enlargement. Cervical stenosis.	Ref [6] pt 4.
6	Severe	*PEX 7*:c [875 T > A]; [875 T > A]	0.6	0.2	9.1	3	Smiles responsively, vocalizes, no intentional movement, bottle/spoon/gastrostomy fed	3 and 14: Ventricular enlargement; frontal hygroma; delayed myelination; abnormal white matter signal intensity; cerebellar atrophy. Cervical canal stenosis.	Ref [6] pt 8
									† 3 yrs 8/12
7	Severe	*PEX 7*:c [875 T > A]; [875 T > A]	0.2	0.1	6.2	35	Eye contact, No intentional movement, gastrostomy fed	1 week. Wide fissura Silvii, no other abnormalities.	
8	Severe	*PEX 7*:c [875 T > A]; [649G > A]	0.2	0.4	7	5	Smiles responsively, vocalizes, scarce spontaneous movement, gastrostomy fed	Not performed.	† 3 yrs 2/12
9	Severe	*PEX 7*:c [875 T > A]; [875 T > A]	1.7	1.4	25.3	11	Smiles responsively, no intentional movement, primarily tube fed	9 and 21: Delayed myelination; abnormal white matter signal intensity; ventricular enlargement; progressive cerebellar atrophy. Relatively narrow cervical canal.	† 2 yrs 7/12
10	Severe	*PEX 7*:c [875 T > A]; [875 T > A]	2.1	1.0	15	6	Responds to sounds, no intentional movement, primarly fed by gastrostomy	Not performed.	† 6 yrs 5/12
11	Severe	*PEX 7*:c [875 T > A]; [875 T > A]	0	0	3.4	Not performed	Smiles responsively, scarce spontaneous movement, primarily tube fed	4: Delayed myelination; wide extra cerebral liquor spaces.	Ref [6] pt 6
									† 7/12
12	Severe	*DHAPAT*:c [924 + 5G > C]; [924 + 5G > C]	0.3	1.2	1.0	Infancy	No intentional movement, gastric tube fed	Not performed.	
13	Mild	*PEX 7*:c [875 T > A];/[854A > G]	5.2	12.1	158.7	4	IQ < 50, walks short distances independently	84: Normal findings.	Ref [6] pt 11
									Diagnosis at age 4 yrs
14*	Mild	*PEX 7*:c [854A > G]; [854A > G]	2.4	6.5	154.1	5	IQ < 50, rides a tricycle	90: Normal findings.	Diagnosis at age 6 yrs
15	Mild	*PEX 7*:c [875 T > A];/?	4	8.5	8.9	24	Autistic features, IQ < 50, walks short distances independently	252: Normal findings.	Ref [6] pt 9
									Diagnosis at age 9 yrs
16	Mild	*PEX 7*:c [875 T > A];/[854A > G]	4.2	6.8	1672	8	Walks short distances unsupported, rides a tricycle, verbal communication, (un)dresses herself	252: Normal findings supratentorial; narrow foramen magnum.	Ref [6] pt 10
									Ref [4]
									Diagnosis at age 9 yrs

♦ Biochemical analysis at time of diagnosis. *Mildest patient in this cohort. Plasmalogens (C16:0DMA/C16:0 and C18:0DMA/C18:0%) measured in RBCs. Phytanic acid measured in plasma. **?**: In two patients only one mutation in *PEX7* was found. Mo: months, nd: not detectable.

†: patient deceased.

Epilepsy occurred in the severe (11/12) and the milder (3/4) phenotype (Table 2). There was a significant difference in age-related onset of seizures between the severe and the milder phenotype (Table 3). In the severe phenotype, ages at onset of epilepsy varied between 4 months and 11 years (median 2.5 years, mean 3.8 years). Patients who survived into school age all developed epilepsy. In the milder phenotype seizures did not occur before age 7 years. Seizure frequency and seizure type varied considerably. Some patients had multiple seizures a day whereas others only had seizures during febrile episodes. Some patients suffered from more than one seizure type. Myoclonic jerks were the most frequent type of seizures reported in patients with the severe phenotype of RCDP. In patients with the milder phenotype brief periods of staring and a frozen facial expression occurred, without the typical 3 Hz spike and slow wave discharges seen on EEG. All treatment in RCDP was aimed at achieving a reasonable quality of life. Since patients experienced discomfort from the frequent seizures, anti epileptic drugs (AED) were prescribed. Sodium valproate was the most commonly used AED (75%). Although generally effective, in patients with the severe phenotype myoclonic epilepsy seizure control was not fully achieved. Parents reported that side effects (drowsiness) of medication were more invalidating to the patient than seizures were on their own. No renal or hepatic side effects were noticed.

**Table 2:** Characteristics of seizures, EEG pattern and EP study results

**Patient**	**Age at epilepsy onset, y**	**Seizure type**	**Seizure frequency**	**Age at EEG examination, y**	**Background pattern first EEG/development of background pattern**	**Focal/multifocal abnormal activity**	**Epileptiform activity**	**Age at EP examination, y**	**BAEP**	**VEP**	**SSEP**
1	2	Myoclonic	Daily	2.0-4.2	M ➔ Mo	Focal	+	3.1 – 4.2	N ➔ N	N ➔ N	-
2	11	Myoclonic; generalized tonic-clonic	Weekly	0.8-11.7	N ➔ S	Focal	+ (≥ 3.8 yrs)	0.8 - 9.1	N ➔ S	N ➔ S	NCR
3	4	1. Fever induced generalized tonic-clonic; 2. (>8.5 yrs) generalized tonic-clonic and atypical absence	1. Sporadic; 2. Weekly	0.8-8.6	N ➔ S	Multifocal	+ (≥ 2.9 yrs)	0.8 - 6.9	N ➔ S	N ➔N	N ➔ M
4	0.3	Tonic; smacking	Daily	0.0-1.3	N ➔ S	Multifocal	+ (≥ 0.5 yrs	0 - 0.3	N ➔ S	N ➔N	-
5	3	Myoclonic; gelastic; atypical absence	Daily	0.9-4.5	M ➔ Mo	Focal ➔ multifocal	+ (≥ 0.9 yrs)	1.3 - 4.5	N ➔ M	N ➔ M	NCR/S
6	2	Myoclonic; status epilepticus	Daily (clustered)	0.2-2.4	N ➔ S	Focal ➔ multifocal	+ (≥ 1.1 yrs) St epilepticus	0.2 - 2.3	N ➔ N	N ➔ N	NCR
7	2	Myoclonic-tonic; gelastic	Daily	0.0-4.0	N ➔ M		+ (≥ 2.3 yrs)	0 -1.8	N	N ➔ N	N
8	0.8	Tonic	Fever induced	0.0-2.2	N			0	N	M	-
9	1	Generalized tonic-clonic	Single, fever induced	0.7-1.7	N ➔ M	Multifocal	+ (≥1.1 yrs)	0.5 - 1.7	N ➔ N	N ➔N	-
10	5	1. Facial myoclonic; 2. tonic	1. Daily; 2. Monthly	5.6	N	Focal	+	-	-	-	-
11				0.3	M		+	0.3	N	N	M
12	10	Myoclonic	Daily	26.7	M	Focal	+	-	-	-	-
13				5.4-7.3	N ➔ N	Focal	+ (≥ 5.4 yrs)	5.4 - 7.3	N ➔ N	N ➔ N	N ➔ N
14	7	Atypical absences	Weekly	6.5-8.3	M		+ (≥ 6.5 yrs)	6.5 - 7.5	N ➔ N	M ➔ N	N ➔ N
15	21	Generalized tonic-clonic	Single	9.3-23.1	M	Focal➔ multifocal	+ (21 yrs)	9.3 - 23.1	N ➔ N	N ➔ M	N
16	20	Atypical absence	Monthly	13.3-29.5	Mo ➔ Mo	Multifocal	+ (≥ 13.3 yrs)	10 - 17	N	N ➔ N	-

N: normal; M: mildly abnormal EEG for age/mildly increased latencies (+2-4 SD), Mo: moderately abnormal EEG background pattern, S: severely abnormal EEG pattern for age/markedly increased latencies (> +4 SD). NCR: no cortical response, y: years.

**Table 3:** Incidence of seizures per age group

**Age, y**	**RCDP cases in this cohort**^**1**^		**Number of cases with epilepsy (%)**^**1**^	
	**Total**	**(Severe/mild)**	**Severe phenotype**	**Mild phenotype**^**3**^
<1	16	(12/4)	2/12 (17%)	0/4 (0%)
1–4	15^2^	(11/4)	9/11 (82%)	0/4 (0%)
4–12	10^2^	(6/4)	6/6 (100%)	1/4(25%)
>12	5^2^	(2/3)	2/2 (100%)	2/3 (67%)

^1^Patients younger at time of analysis and patients deceased are excluded. ^2^One patient died at age <1 yrs; 4 patients died at age 1–4; 2 patients died at age 4–12 yrs. ^3^Patients with the milder phenotype were diagnosed after age 4 years.

y: years.

**Figure 2:**
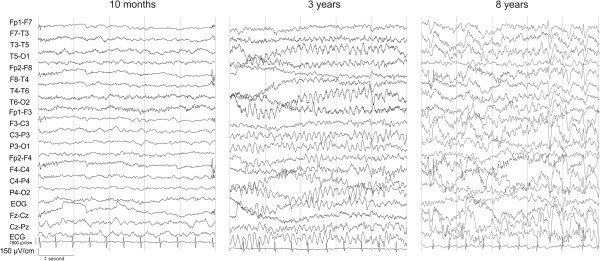
**Change in EEG pattern in a patient with the severe phenotype of RCDP.** EEG recordings of patient 3. Normal for age at 10 months. Development of background pattern at 3 years. Deterioration of background pattern and runs of spike wave complexes in the frontal regions at 8 years.

A total of 63 EEGs were conducted. Interictal background EEG rhythms were initially normal in 10/16. Non-epileptiform focal or multifocal abnormal activity occurred in 12/16. Deterioration to slow and less organized patterns (Figure 2), with diminished (to absent) reactivity on stimuli occurred in 7 patients with the severe phenotype. Patients with the severe phenotype that did not show epileptiform discharges on EEG were under the age of 1 year. During EEG recording, EEG seizure discharges correlating with clinical seizure activity was seen in 7/14.

Evoked potential study results are summarized in Table 2. A total of 39 VEPs were performed in 10 with the severe and 4 patients with the milder phenotype. Follow up examinations were performed in 8 with the severe and 3 patients with the milder phenotype. In 12/14 the initial latencies were normal. Deterioration occurred in 3 (2 patients with the severe phenotype and 1 patient with the milder phenotype). All patients underwent lens extraction because of bilateral congenital cataracts. There was no relationship between the time of lens extraction and functional integrity of the visual system as measured by VEP.

A total of 34 BAEPs are available from 10 with the severe and 3 patients with the milder phenotype, with follow up in 10. Initial BAEPs were all normal. In 4/7 patients with the severe phenotype in whom follow up was performed, central prolonged conduction times at BAEP occurred in time. In patients with the milder phenotype, latencies were normal.

Eighteen SSEPs were recorded in 6 with the severe and 3 with the milder phenotype. In the patients with the milder phenotype latencies were normal. Two patients with the severe phenotype had normal conduction times. In 2 patients latencies were increased and in another 2 no cortical responses could be detected. These patients also showed stenosis of the cervical spinal canal on MRI, even though there were no signs of myelum compression [6].

## Discussion

In this report we charted the long term follow-up of a cohort of 16 patients with RCDP and correlated the outcome to the severity of the genetic and metabolic defects (the largest series so far). Patients with both the mild and the severe phenotype are represented and biochemical and genetical data are available of all patients.

The clinical neurologic presentation of patients at the severe end of the RCDP spectrum is quite homogenous, with profound epilepsy, spasticity and near absent development. Decay in neurologic skills is not typical, although interaction with the surrounding is less in periods of illness and frequent seizures [15]. Less is known about the disease course of patients with the milder phenotype of the disorder [4,6,16,17]. In this cohort, 4 patients with the milder clinical and biochemical phenotype are represented. They attain more advanced developmental skills than patients with the severe phenotype. The 2 eldest patients become less ambulant with time. Patients with the milder phenotype have significant higher levels of plasmalogens in red blood cells. The importance of plasmalogens in the neurological profile is further emphasized by the fact that the patient with a single enzyme deficiency in plasmalogen biosynthesis is comparable to the patients with the severe variant of RCDP type 1.

In order to unravel the effects of plasmalogen deficiency in vivo, several mouse models for both the severe and milder variant of RCDP type 1, 2 and 3 have been generated. As in the patients, the severity of the clinical phenotype in mice is related to the residual capacity in plasmalogen biosynthesis. Eye, brain and bone abnormalities resemble the abnormalities seen in patients with RCDP. Growth is impaired. Life expectancy appears to be dependent on the ability to survive a critical period after birth in *Pex7*^-/-^, but is normal in the *Pex7*^*neo/neo*^ mice [18-21]. Epilepsy has not been mentioned in the mice.

Seizures represent a significant clinical problem in RCDP. Our results on the occurrence of seizures are in agreement with earlier studies [16]. However, previous studies have not considered the impact of the severity of biochemical defects and specific mutations in relation to epilepsy [22,23]. We found that epilepsy occurs frequently in RCDP patients irrespective of genotype or phenotype. However, a clear distinction between patients with the mild and the severe phenotype existed in the age at onset of epilepsy. This implicates that there is a relation between the development of seizures and the amounts of residual plasmalogens. This is in agreement with the current knowledge about plasmalogen distribution and function since they are known to be abundant in gray matter where they play an active metabolic role [8,24]. We were particularly interested in the latencies of evoked potentials as a reflection of myelination, since in humans plasmalogens constitute the majority of total phospholipids in the white matter and signal abnormalities on MRI are present in patients with the severe, but not the milder phenotype of RCDP [6,8]. We found that 93% of VEP latencies were initially normal for age and that deterioration occurred in 31% of patients with both the severe and mild phenotype. Because both mouse models for severe RCDP (*PEX7*^*-/-*^ and *GNPAT*^*-/-*^) show hypoplasia of the optic nerves [25], we are quite surprised that VEPs were relatively spared and that there is no obvious difference between patients with the severe and mild phenotype. BAEPs and SSEPs are more likely to be abnormal in the patients with the severe phenotype. This indicates a loss of integrity of the brainstem auditory system and the lemniscal and thalamocortical pathways in the brain in some of the patients with the severe phenotype of RCDP. Indeed, both delayed myelination and dysmyelination are described in RCDP patients [6,26,27]. Currently we do not have an explanation why some of our most severely affected individuals have normal evoked potentials.

In conclusion, the age related occurrence of epilepsy and the severe, but variable defects in motor and cognitive functioning of patients, illustrates the importance of plasmalogens for normal neurodevelopment. Evoked potentials are more likely to become abnormal in patients with low amounts of plasmalogens, but have no predictive value in single cases.

## Consent

Written informed consent for publication of Figure 1 was given by the parents of the patient.

## Competing interest

The authors declare that they have no competing interests.

## Authors’ contributions

AB: Design of the study, interpretation of data, drafting manuscript. JK: Interpretation of EEGs and EP studies, revising the manuscript. HW: Providing biochemical and genetic information, revising the manuscript. PB: Interpretation of data, revising the manuscript. BP: Guarantor, design of the study, interpretation of data, revising manuscript. All authors read and approved the final manuscript.
